# Hemorrhagic bullous lesions in Henoch-Schönlein purpura: a case report and review of the literature

**DOI:** 10.1186/s12887-018-1117-8

**Published:** 2018-05-10

**Authors:** Hung-Wen Su, Chiu-Yu Chen, Yee-Hsuan Chiou

**Affiliations:** 1 Division of Pediatric Allergy, Immunology and Rheumatology, Department of Pediatrics, Kaohsiung Veterans General Hospital, No. 386, Dazhong 1st Road, Zuoying District, Kaohsiung City, 813 Taiwan; 2 School of Medicine, National Yang-Ming University, Taipei, Taiwan

**Keywords:** Hemorrhagic bullae, Henoch-Schönlein purpura, Leukocytoclastic vasculitis, Case report

## Abstract

**Background:**

Henoch-Schönlein purpura (HSP) is a common vasculitis in childhood characterized by purpura, arthritis, abdominal pain and renal involvement. However, bullous HSP is a rare cutaneous manifestation, and a few cases have been reported.

**Case presentation:**

Herein, we report a 15-year-old male with bullous HSP who presented with severe abdominal pain and hemorrhagic bullous lesions over his lower extremities. He was treated with corticosteroid, after which the symptoms improved dramatically. No recurrence was noted after follow-up, though scarring was present. We also reviewed the literature related to bullous HSP and identified 39 cases, most of whom were treated with corticosteroids.

**Conclusion:**

Clinicians should be aware of the atypical types of HSP, including bullous HSP. Most patients with bullous HSP have a good prognosis.

## Background

Henoch-Schönlein purpura (HSP) is one of the most common forms of vasculitis in childhood. It is characterized by cutaneous purpura, arthritis, gastrointestinal (GI) symptoms, and renal involvement. Classification criteria of HSP were recently proposed by EULAR/PRINTO/PRES. They include purpura (commonly palpable and in crops) or petechiae, predominantly over the lower limbs and with at least one of the four following criteria: (1) diffuse abdominal pain; (2) biopsy revealing leukocytoclastic vasculitis with predominant IgA deposition; (3) arthritis or arthralgia; and (4) renal involvement, including proteinuria or hematuria [1]. HSP is a potentially self-limiting illness, but recurrence has been reported in approximately 30% of patients. The incidence of HSP ranges from 6.7 to 22 per 100,000 children [2–6]. Vanesa et al. reported that the most common symptoms of recurrence episodes in the first months after the first episode are abdominal pain and joint manifestations [6]. HSP occurs mostly between the ages of 5 and 15, and the reported mean ages range from 4 to 7 years [2, 3]. The dermatologic manifestations are usually palpable purpura and petechiae. Some lesions, such as urticarial or erythematous maculopapular lesions, may also be found. The skin rash is usually present on pressure-bearing sites, especially the lower extremities and buttocks. Hemorrhagic bullae and vesicles are unusual manifestations. In this study, we present a 15-year-old male with bullous HSP and review 39 cases reported in the literature since 1985.

## Case presentation

**Fig. 1:**
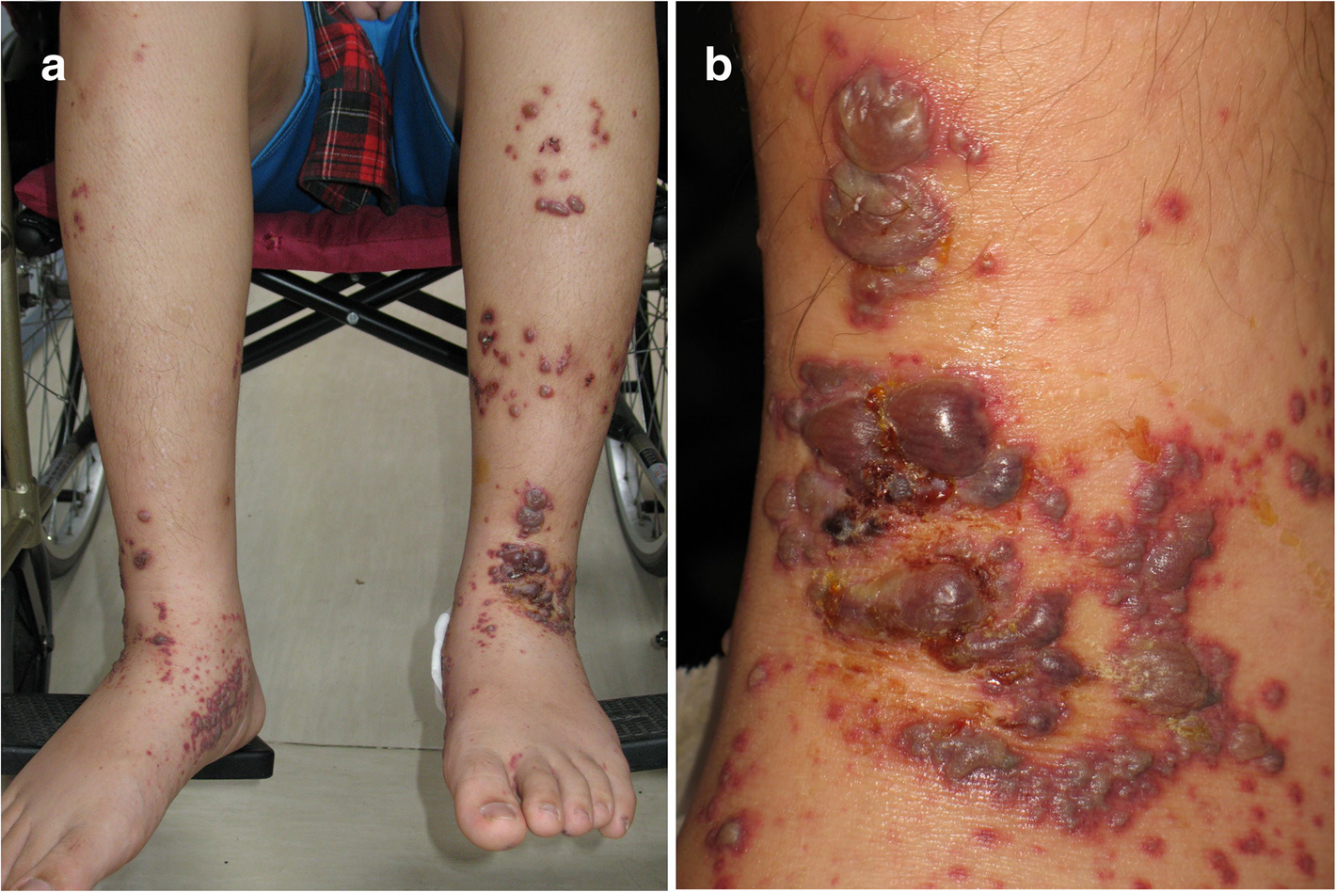
**a** Purpura and bullae over both legs. **b** Hemorrhagic bullae, purpura and crust lesions over his left foot

A previously healthy 15-year-old Chinese boy presented with abdominal pain for 5 days, followed by maculopapular purpuric lesions over both lower extremities 2 days later. These lesions progressed to vesicles and bullae, and he was then admitted to our hospital. His medical history was unremarkable, and no upper airway symptoms were noted before this episode. No family history of systemic disease nor HSP was noted. A physical examination on admission revealed a body temperature of 36.8 °C, pulse rate of 96/min, respiratory rate of 20/min, and blood pressure of 130/83 mmHg. Palpable purpura, hemorrhagic bullae, vesicles, and crust over bilateral legs and feet were noted, especially in his left leg (Fig. 1). The bullae and vesicles ranged in size from 2 mm to 20 mm in diameter. His buttocks, palms, trunk and face were spared.

**Fig. 2:**
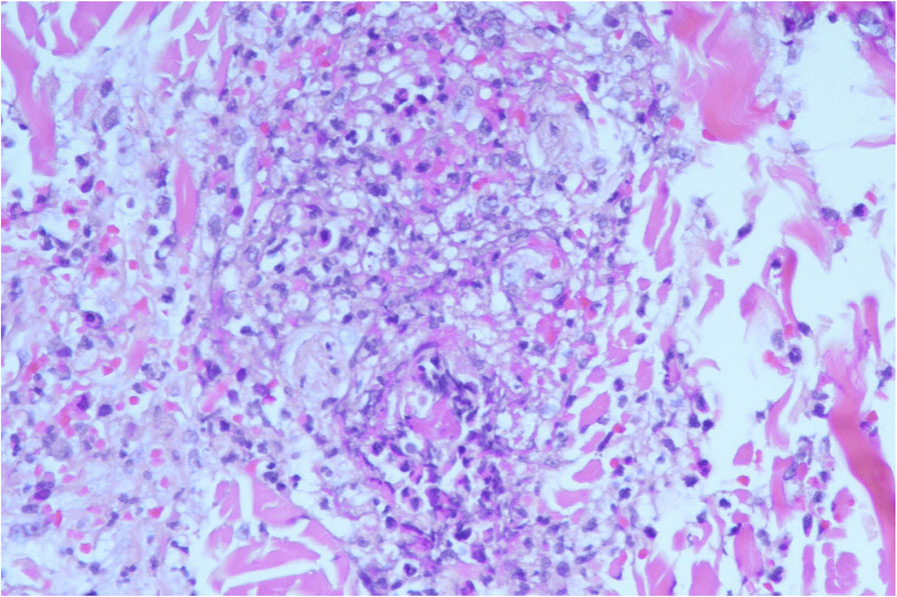
Skin biopsy showed endothelial swelling with fibrinoid necrosis of small venules surrounded by intense neutrophilic infiltration, erythrocyte extravasation and neutrophilic karyorrhectic debris (hematoxylin and eosin stain, × 400)

Laboratory data revealed a white blood cell count of 17,860/μL (reference range, 4000–1,0500/μL) with normal differentiation, hemoglobin 16.0 g/dL (reference range, 12.5–16.1 g/dL), platelet count 326,000/μL (reference range, 15,000–40,000/μL), C-reactive protein 7.3 mg/dl (reference range, 0.04–0.8 mg/dl), blood urea nitrogen 10 mg/dL (reference range, 7–18 mg/dL), and creatinine 0.8 mg/dL (reference range, 0.5–1.2 mg/dL). The prothrombin time and activated partial thromboplastin time were normal, and serum levels of IgG, IgA, IgM, ANA, P-ANCA, C-ANCA, antistreptolysin O, complement 3 (C3), and C4 were also within normal limits. The erythrocyte sedimentation rate was 3 mm/hour (reference range, 0–15 mm/hour) on the third day of admission. Urine analysis showed no hematuria or proteinuria, and stool analysis showed no occult blood. A throat swab culture and viral isolation were negative. Serologic titers for cytomegalovirus, herpes simplex virus, and varicella-zoster virus were also negative. A skin biopsy of hemorrhagic and purpuric lesions was performed on the third day of admission and disclosed leukocytoclastic vasculitis in the epidermis and extended to the superficial and deep dermis (Fig. 2). Polymorphonuclear neutrophils also infiltrated in the subcutaneous tissue. An immunofluorescence examination showed no deposition of IgA, IgG, or IgM. However, deposition of C3 was noted around the vessel walls in the dermis. The pathological findings were consistent with HSP.

The patient was initially given oxacillin for suspected cellulitis. Hydrocortisone 10 mg/kg/day was administered intravenously for 3 days rather than oral corticosteroids due to severe abdominal pain, after which the dosage was tapered. On the second day of admission after we administered corticosteroid, his severe abdominal pain persisted. Hence, abdomen computed tomography (CT) was performed to rule out other acute abdomen diseases. It revealed only multiple bulging lymph nodes over the right upper quadrant. Hydrocortisone was applied for 7 days, which was then shifted to oral prednisolone for 8 weeks. The abdominal pain improved, and the bullous lesions resolved within 1 week. Some scar formation was noted after that. Urine analysis 3 months later revealed normal findings. No recurrence was noted in the following 3 months. After follow-up for more than one year, no recurrence of skin manifestation was noted, but the scarring over his left anterior leg and right posterior leg was still present.

## Discussion

Henoch-Schönlein purpura is the most common type of vasculitis during childhood. It is an immune-mediated vasculitis which is associated with IgA deposition, but its pathogenesis is unknown. Our patient met the criteria of HSP. Typical skin manifestations of HSP include palpable purpura and petechiae. However, erythematous maculopapules and urticarial lesions have also been reported. Hemorrhagic bullous and ulcerative lesions are rare in pediatric patients, and the diagnosis of bullous HSP can be challenging. The bullous lesions that occur in children include erythema multiforme, toxic epidermal necrolysis, bullous congenital ichthyosiform erythroderma, epidermolysis bullosa, bullous pemphigoid, pemphigus, linear IgA dermatosis, bullous systemic lupus erythematosus, bullous impetigo, eczema herpeticum, and dermatitis herpetiformis. However, bullous lesions are relatively common in adult HSP, reportedly occurring in 16% to 60% of cases [7, 8].

We searched PubMed for studies published from 1985 to February 2016, using the keywords “bullous” and “Henoch-Schönlein purpura”. We also scanned the references of all search results for additional studies and identified only 39 patients (including our patient) who presented with hemorrhagic bullae (Table 1). One patient reported by Abdel-al et al. was excluded due to missing data [4]. Table 2 lists the main characteristics of these 38 patients with bullous HSP. The male to female ratio was 1:1, and the mean age at the diagnosis of bullous HSP was 8.2 years (range, 3–15 years). The most common systemic symptom was joint involvement (63.2%), followed by gastrointestinal involvement (60.5%), and renal involvement (39.5%). Orchitis is a complication of HSP [2], and one patient who had scrotum pain underwent surgery [9]. Recurrence only occurred in two patients.

**Table 1:** Summary of cases of Henoch-Schönlein purpura with hemorrhagic bullous lesions

Reference	Sex	Age	Lesion location	Systemic symptoms	Skin IF: IgA	Therapy	Outcome
Garland et al. [24]	M	5	Elbow, thigh, buttocks, and perioral region	J, GI, R	NR	Bed rest	Resolved
Bari et al. [25]	F	7	Shins	J	Negative	None	Resolved
Abdel-al et al. [4]	NR	NR	NR	NR	NR	NR	NR
Wananukul et al. [26]	M	5	Pinnas, hard palate, gums, hands, buttocks and legs	J, GI, R	Positive	PSL	Resolved
Saulsbury [27]	F	7	Feet and ankle	GI	Positive	PRD	Resolved
	F	3	Legs and feet	GI, R	ND	PRD	Hematuria persisted
Kobayashi et al. [13]	M	10	Shins	J, GI	Negative	Bed rest	Resolved
Liu et al. [28]	F	7	Buttocks and lower extremities	J	Negative	HC	Resolved
	M	6	Buttocks and lower extremities	J, GI	Negative	HC	Resolved
Ishii et al. [29]	M	4	Face, pinnus, buttocks, hands and legs	J, GI	Positive	PSL, MTP	Resolved
Lueng et al. [12]	M	8	Ankles and feet	J, GI	NR	None	Resolved
Chan et al. [22]	M	14	Lower extremities	GI	Negative	Colchicine	Resolved
Korver et al. [30]	F	10	Legs and right foot	NR	NR	NR	Resolved
Aydinoz et al. [31]	F	4	Lower extremities	J	NR	None	Hyperpigmentation
Abdul-Ghaffar et al. [32]	M	10	Lower legs, feet, and hands	J, GI, R	Positive	PSL	Resolved
Júnior et al. [33]	F	9	Face, arms, buttocks, hands and feet	GI	Positive	PRD	Ulcers hyperpigmentation
	F	9	Feet	J, R	ND	PRD	Resolved
	F	6	Feet	J	Positive	None	Hyperpigmentation
Kausar et al. [34]	M	6	Lower extremities, trunk, buttocks, arms and genitalia	J, R	NR	antibiotics	Resolved
Maguiness et al. [23]	M	8	Hands and feet	J, GI	Positive	PSL	Recurrence
	F	15	Lower extremities	No	Negative	Fluocinonide	Resolved
	M	8	Abdomen, lower extremities	J, GI	Positive	Betamethasone	NR
	M	8	Lower extremities	No	ND	None	NR
	F	11	Lower extremities	GI	Positive	None	NR
	F	10	Lower extremities	GI	ND	MTP	NR
den Boer et al. [17]	M	6	Arms and legs	GI, R	Positive	PRD	Scarring
	M	10	Legs	R	ND	PSL	Scarring
Trapani et al. [19]	F	9	Buttocks, legs, arms, hands and face	J, GI, R	ND	MTP, PRD	Hyperpigmentation and scarring
	M	11	Buttocks, legs, feet and ankles	J	ND	None	Resolved
	F	7	Buttocks, legs, feet and ankles	J, GI	ND	MTP, PRD, AZA	Resolved
Park et al. [11]	F	3	Legs and feet	J, GI, R	ND	MTP, PRD	Scarring, hematuria
Parikh [35]	M	14	Lower extremities	GI	NR	PSL	Resolved
Raymond et al. [36]	F	9	Feet and ankles	J, R	ND	PSL	Resolved
Kocaoglu et al. [37]	F	4	Lower extremities	J, GI, R	NR	PSL	Resolved
Mehra et al. [20]	F	9	Lower extremities, trunk, buttocks, and ear	GI, R	Positive	DXM, PSL, MTP, AZA	Resolved
Gration et al. [9]	M	3	Lower limbs, buttocks, forearms and elbows	J, S	NR	None	Recurrence
Chen et al. [10]	F	14	Arms, legs, abdomen, and buttocks	J, R	Positive	PRD, Dapsone	Scarring and proteinuria
Hooper et al. [38]	M	9	Lower extremities, buttock, and arms	J, R	NR	Eumovate	Resolved
Present case	M	15	Legs and feet	GI	Negative	HC, PSL	Scarring

*J* joint, *GI* gastrointestinal involvement, *R* renal involvement, *S* scrotum pain, *NR* not reported, *ND* not done, *PRD* prednisone, *PSL* prednisolone, *HC* hydrocortisone, *MTP* methylprednisolone, *DXM* dexamethasone, *AZA* azathioprine, *IF* immunofluorescence

**Table 2:** Main characteristics of the 38 patients with bullous HSP

	Children	Percentage (%)
Sex		
Male	19	50
Female	19	50
Male/female ratio	1:1	
Age (years)		
Mean± SD	8.2± 3.32	
Systemic symptoms		
Joint involvement	24	63.2
GI involvement	23	60.5
Renal involvement	15	39.5
Scrotum involvement	1	2.6
No systemic symptom	1	2.6
Skin biopsy	19	50
Positive of IgA deposition	12	31.6
Negative of IgA deposition	7	18.4
Therapy		
Systemic corticosteroid use	22	57.9
Topical corticosteroid use	3	7.9
Azathioprine	2	5.3
Colchicine	1	2.6
Dapsone	1	2.6
No treatment	9	23.7
No reported	1	2.6
Outcome		
Resolved	22	57.9
Hyperpigmentation	4	10.5
Scarring	6	15.8
Hematuria	2	5.3
Proteinuria	1	2.6
Recurrence	2	5.3
No reported	5	13.2

HSP is a leukocytoclastic vasculitis that affects small vessels, and the characteristic histological finding is neutrophil infiltration around papillary and dermal vessels. The deposition of IgA (especially IgA_1_) and C3 was commonly noted in direct immunofluorescence studies. Not all of the patients had IgA deposition, however, and of the 19 patients who underwent skin biopsies, only 12 (63%) had IgA deposition. The timing of biopsy may affect the finding of IgA deposition, and early biopsy is necessary to make the diagnosis. Immunoreactants, including IgA and C3, are destroyed within 48 h [10]. Our patient was also negative for IgA deposition, but positive for C3 deposition around the vessel walls in the dermis. Skin biopsy was performed after 48 h of appearance in our patient, so that might have led to the false-negative result on direct immunofluorescence. The leukocytoclastic vasculitis is usually limited to the upper layer of the dermis, but one study reported that the leukocytoclastic vasculitis extended to the deeper layer of the dermis and resulted in scar formation [11]. Our patient had scarring, and histopathology showed the deeper layer of the dermis and subcutaneous tissue had been affected.

Most patients had lesions over their lower extremities and buttocks, and 4 of the 38 patients had lesions over their faces and even their ears. Leung et al. reported that the most severe lesions were commonly observed under points of maximal pressure, suggesting that pressure is a factor in the pathogenesis of bullous HSP [12]. Kobayshi et al. reported that matrix metallopeptidase-9 (MMP-9, gelatinase) is secreted by polymorphonuclear neutrophils, which can then cause the formation of blisters by degrading type VII collagen in basement membranes. This may also be an important factor in the pathogenesis [13].

With regards to therapy, there is no consensus on the best treatment for bullous HSP. No randomized trials have been conducted due to the rarity of bullous HSP. Some studies reported that corticosteroids may reduce the severity of abdominal pain and the risk of developing persistent renal disease, but it is not reported to prevent recurrence [2, 14–16]. den Boer et al. suggested that early prednisolone treatment may reduce the severity and extent of the bullous lesions [17], and Park et al. suggested that the anti-inflammatory effect of corticosteroids may be useful in treating bullous HSP [18]. In our review, 22 patients (58%) received corticosteroid treatment, and only one had recurrence. More studies are needed to confirm the effect of corticosteroids in the management of the cutaneous lesions in HSP. In our patient, the abdominal pain improved dramatically, and the skin lesions also improved within days after hydrocortisone treatment. We started corticosteroid therapy with the aim of reducing the severity of his abdominal pain as well as the skin manifestations. No recurrence was noted.

Two studies reported the use of azathioprine with corticosteroids in two patients due to uncontrolled skin lesions and progressive heavy proteinuria [19, 20]. One study reported that dapsone, an antileprotic drug, was useful in treating HSP [21], and Chen et al. reported the use of dapsone to wean a patient off prednisone [10]. Colchicine also has been used in one patient for a patient who was as a chronic hepatitis B carrier [22]. Nine patients had no treatment, and their lesions gradually resolved. Avoiding infection, trauma and the use of antibiotic ointment or protective dressings may also play an important role in bullous HSP, as in other bullous diseases [17].

HSP is usually a self-limiting disease, but one-third of patients will experience one or more episodes of recurrence of symptoms [2]. In our review, only two patients had recurrence [9, 23]. The long-term prognosis of HSP seems to depend on the severity of renal involvement [2]. Only three of the patients in our review had renal involvement, including persistent hematuria and proteinuria after treatment [2, 10, 11]. Most of the studies reported that the lesions resolved, but a few patients had scarring and pigmentation. Our patient had no recurrence of skin manifestations or hyperpigmentation, but scarring was still present after follow-up.

## Conclusion

Hemorrhagic bulla is a rare cutaneous manifestation in children with HSP. This report aims to raise awareness of this atypical type of HSP. The use of corticosteroids may be beneficial for patients with bullous HSP. In general, most patients with bullous HSP have a good prognosis.

## Abbreviations

ANA: Antinuclear antibodies
ANCA: Antineutrophil cytoplasmic antibodies
CT: Computed tomography
EULAR: European League Against Rheumatism
HSP: Henoch-Schönlein purpura
IgA: Immunoglobulin A
IgG: Immunoglobulin G
IgM: Immunoglobulin M
PRES: Paediatric Rheumatology European Society
PRINTO: Paediatric Rheumatology International Trials Organisation

## References

[1] Ozen S, Pistorio A, Iusan SM, Bakkaloglu A, Herlin T, Brik R (2010). EULAR/PRINTO/PRES criteria for Henoch-Schonlein purpura, childhood polyarteritis nodosa, childhood Wegener granulomatosis and childhood Takayasu arteritis: Ankara 2008. Part II: final classification criteria. Ann Rheum Dis.

[2] Saulsbury FT (1999). Henoch-Schonlein Purpura in children report of 100 patients and review of the literature. Medicine.

[3] Trapani S, Micheli A, Grisolia F, Resti M, Chiappini E, Falcini F (2005). Henoch Schonlein Purpura in childhood: epidemiological and clinical analysis of 150 cases over a 5-year period and review of literature. Semin Arthritis Rheum.

[4] Abdel-Al YK, Hejazi Z, Majeed H (1990). Henoch-Schönlein purpura in Arab children: analysis of 52 cases. Trop Geogr Med.

[5] Gardner-Medwin JMM, Dolezalova P, Cummins C, Southwood TR (2002). Incidence of Henoch-Schonlein purpura, Kawasaki disease, and rare vasculitides in children of different ethnic origins. Lancet.

[6] Vanesa CR, José LH, Francisco OS, Javier L, Natalia PF, Maria CGV (2016). Relapses in patients with Henoch–Schönlein purpura: analysis of 417 patients from a single center. Medicine.

[7] Cream JJ, Gumpel JM, Peachey RDG (1970). Schönlein-Henoch purpura in the adult. A study of 77 adults with anaphylactoid or Schönlein-Henoch purpura. Q J Med.

[8] Tancrede-Bohin E, Ochonisky S, Vignon-Pennamen M-D, Flageul B, Morel P, Rybojad M (1997). Schönlein-Henoch purpura in adult patients: predictive factors for IgA glomerulonephritis in a retrospective study of 57 cases. Arch Dermatol.

[9] Gration B, Osakwe E. Self-limiting recurrent bullous Henoch-Schonlein purpura with lupus anticoagulant. BMJ Case Rep. 2015; 10.1136/bcr-2014-205436. 10.1136/bcr-2014-205436 PMC4307072 25604501

[10] Chen CB, Garlapati S, Lancaster JD, Zinn Z, Bacaj P, Patra KP (2015). Bullous Henoch-Schönlein Purpura in Children. Cutis.

[11] Park SE, Lee JH (2011). Haemorrhagic bullous lesions in a 3-year-old girl with Henoch-Schölein purpura. Acta Paediatr.

[12] AKC L, WLM R (2006). Hemorrhagic bullous lesions in a child with Henoch-Schönlein Purpura. Pediatr Dermatol.

[13] Kobayashi T, Sakuraoka K, Iwamoto M, Kurihara S (1998). A case of anaphylactoid purpura with multiple blister formation: possible pathophysiological role of gelatinase (MMP-9). Dermatology.

[14] Ronkainen J, Koskimies O, Ala-Houhala M, Antikainen M, Merenmies J, Rajantie J (2006). Early prednisone therapy in Henoch-Schonlein purpura: a randomized, double-blind, placebo-controlled trial. J Pediatr.

[15] Weiss PF, Feinstein JA, Luan X, Burnham JM, Feudtner C (2007). Effects of corticosteroid on Henoch-Schonlein purpura: a systematic review. Pediatrics.

[16] Weiss PF, Klink AJ, Localio R, Hall M, Hexem K, Burnham JM (2010). Corticosteroids may improve clinical outcomes during hospitalization for Henoch-Schonlein purpura. Pediatrics.

[17] den Boer SL, Pasmans SG, Wulffraat NM, Ramakers-Van Woerden NL, Bousema MT (2010). Bullous lesions in Henoch Schönlein Purpura as indication to start systemic prednisone. Acta Paediatr.

[18] Park SJ, Kim JH, Ha TS, Shin JI (2011). The role of corticosteroid in hemorrhagic bullous Henoch Schönlein purpura. Acta Paediatr.

[19] Trapani S, Mariotti P, Resti M, Nappini L, Martino M, Falcini F (2010). Severe hemorrhagic bullous lesions in Henoch Schonlein purpura: three pediatric cases and review of the literature. Rheumatol Int.

[20] Mehra S, Suri D, Dogra S, Gupta A, Rawat A, Saikia B (2014). Hemorrhagic bullous lesions in a girl with Henoch Schönlein purpura. Indian J Pediatr.

[21] Iqbal H, Evans A (2005). Dapsone therapy for Henoch-Schönlein purpura: a case series. Arch Dis Child.

[22] Chan KHN, Tang WYM, Lo KK (2007). Bullous lesions in Henoch-Schönlein purpura. Pediatr Dermatol.

[23] Maguiness S, Balma-Mena A, Pope E, Weinstein M (2010). Bullous Henoch-Schonlein purpura in children: a report of 6 cases and review of the literature. Clin Pediatr.

[24] Garland JS, Chusid MJ (1985). Henoch-Schöenlein purpura: association with unusual vesicular lesions. Wis Med J.

[25] Bari M, Cohen BA (1990). Purpuric vesicular eruption in a 7-year-old girl. Arch Dermatol.

[26] Wananukul S, Pongprasit P, Korkij W (1995). Henoch-Schonlein purpura presenting as hemorrhagic vesicles and bullae: case report and literature review. Pediatr Dermatol.

[27] Saulsubury FT (1998). Hemorrhagic bullous lesions in Henoch-Schönlein purpura. Pediatr Dermatol.

[28] Liu PM, Bong CN, Chen HH, Huang YC, Huang CC, Yang KD (2004). Henoch-Schönlein purpura with hemorrhagic bullae in children: report of two cases. J Microbiol Immunol Infect.

[29] Ishii Y, Takizawa T, Arakawa H, Saga R, Mochizuki H, Tokuyama K (2005). Hemorrhagic bullous lesions in Henoch-Schönlein purpura. Pediatr Int.

[30] Korver A, Moons P (2007). Diagnostic image (329). A girl with haemorrhagic bullae. Ned Tijdschr Geneeskd.

[31] Aydinoz S, Karademir F, Suleymanoglu S, Ozkaya H, Ersen A, Gocmen I (2007). An unusual manifestation of Henoch-Schonlein purpura: haemorrhagic bullous lesions. N Z Med J.

[32] Abdul-Ghaffar S, Chan SK, Burrows NP (2007). Haemorrhagic bullae in a child with Henoch-Schönlein purpura. Br J Dermatol.

[33] Júnior CR, Yamaguti R, Ribeiro AM, Melo BA, Campos LA, Silva CA (2008). Hemorrhagic vesicle-bullous lesions in Henoch-Schönlein purpura and review of literature. Acta Reumatol Port.

[34] Kausar S, Yalamanchili A (2009). Management of haemorrhagic bullous lesions in Henoch-Schonlein purpura: is there any consensus?. J Dermatolog Treat.

[35] Parikh K (2012). A 14-year-old boy with bullous lesions. Pediatr Ann.

[36] Raymond M, Spinks J (2012). Bullous Henoch Schonlein purpura. Arch Dis Child.

[37] Kocaoglu C, Ozturk R, Unlu Y, Akyurek FT, Arslan S (2013). Successful treatment of hemorrhagic bullous Henoch-Schönlein purpura with oral corticosteroid: a case report. Case Rep Pediatr.

[38] Hooper JE, Lee C, Hindley D (2016). Case report: bullous Henoch-Schönlein purpura. Arch Dis Child.

